# Personalized Dual Antiplatelet Therapy in Acute Coronary Syndromes: Striking a Balance Between Bleeding and Thrombosis

**DOI:** 10.1007/s11886-023-01892-9

**Published:** 2023-06-01

**Authors:** Jonathan Shpigelman, Anastasia Proshkina, Michael J. Daly, Dermot Cox

**Affiliations:** 1 School of Medicine, Royal College of Surgeons in Ireland, Dublin, Ireland; 2 Department of Cardiology, Connolly Hospital, Blanchardstown, Dublin, Ireland; 3 School of Pharmacy and Biomolecular Sciences, Royal College of Surgeons in Ireland, Dublin, Ireland

**Keywords:** Precision medicine, Personalized medicine, Aspirin, Dual antiplatelet therapy, Percutaneous Coronary intervention, Acute coronary syndrome

## Abstract

**Purpose of Review:**

Dual antiplatelet therapy (DAPT)—aspirin in conjunction with a P2Y_12_ inhibitor—is the cornerstone of managing patients with acute coronary syndromes post-revascularization, but the clinical response is highly variable, with potentially devastating consequences. Herein, we review the mechanisms underpinning said variability and explore emerging approaches to normalizing therapeutic benefit.

**Recent Findings.:**

The potent P2Y_12_ inhibitors, prasugrel and ticagrelor, exhibit minimal inter-individual variability, replacing clopidogrel in DAPT and achieving greater rates of therapeutic response. However, these benefits decline in later phases when bleeding risk begins to supersede that of ischemia. Guided de-escalation of P2Y_12_ inhibition as well as shortening DAPT duration have emerged as strategies that retain antithrombotic efficacy while reducing bleeding risk. Aspirin is the other component of DAPT but is also used in isolation for secondary prevention of thrombotic disease. In contrast to the P2Y_12_ inhibitors, genetic influences on aspirin non-response appear to be outweighed by a triad of clinical factors: non-adherence, enteric aspirin use, and inappropriate dosing according to bodyweight and BMI.

**Summary:**

Multiple de-escalation strategies for DAPT have been shown to mitigate bleeding risk, but it remains unclear which approach is ideal, necessitating head-to-head investigations to determine which exhibits the most favorable cost-to-benefit ratio. However, there is likely a role for more than one approach in clinical practice, depending on patient risk profile. Our approach to aspirin use is also in need of reassessment: strategies to improve adherence, avoidance of enteric aspirin in cardiac patients, and dose adjustment according to bodyweight and/or BMI are all likely to improve rates of therapeutic response. Moreover, platelet function testing may have a role in identifying patients expected to benefit from primary prophylactic aspirin.

**Supplementary Information:**

The online version contains supplementary material available at 10.1007/s11886-023-01892-9.

## Introduction

Like most drugs, antiplatelet agents are dosed according to average population requirements. While this strategy may have little impact in most clinical scenarios, certain antiplatelet agents have notoriously variable inter-individual efficacies, meaning that many patients will be inadvertently under- or overdosed. Some patients may even exhibit a complete lack of response, whereby no reasonable dose could confer benefit, necessitating an alternative therapy altogether. Consequently, treatment failure with these medications is common and carries potentially devastating consequences. For instance, aspirin non-response is associated with a four-fold increase in cardiovascular events [1] and over a two-fold increase in the risk of stroke [2]. However, we cannot universally increase the standard dosing to mitigate these risks as any benefit must be balanced by the associated increased risk of bleeding, especially hemorrhagic stroke, in patients who are “strong responders.” To reconcile these opposing considerations, it is critical to prescribe antiplatelet agents in an individualized manner that achieves the optimal therapeutic effect. This approach, known as precision medicine, can be achieved through the cumulative consideration of a patient’s genetic profile (pharmacogenomics), clinical characteristics, concomitant interacting medications, and lifestyle factors. Alternatively, patients may be phenotypically stratified using functional assays that directly assess drug responsiveness which, in theory, circumvents the need to account for all factors that may influence a drug’s efficacy; however, phenotyping has its own challenges and is unlikely to be an end-all solution. The best-known example is the international normalized ratio (INR), a functional assay of coagulation used to guide appropriate warfarin dosing. However, due to warfarin’s delayed onset of action and its narrow therapeutic range, the process of dose titration can potentially take months [3]. Pharmacogenomics, along with other relevant patient factors, can be used algorithmically to optimize the initial dosing and achieve a more timely target INR [3]. Therefore, there appears to be a role for both approaches, depending on the specific context. Regarding antiplatelet agents, there are many functional assays, but they remain poorly standardized and their results are often incongruous [4, 5]. Hence, improved platelet function tests (PFTs) may prove to be indispensable tools for drugs whose efficacies are highly multifactorial, such as aspirin.

These strategies to correct for inter-individual variability are particularly relevant in patients with acute coronary syndromes (ACS) treated with percutaneous coronary intervention (PCI) and stent implantation, who are typically managed with 1 year of dual antiplatelet therapy (DAPT) post-revascularization. The prototypical DAPT regimen consists of aspirin and the P2Y_12_ inhibitor, clopidogrel, both of which have been associated with considerable inter-subject variability. However, clopidogrel has now been virtually phased out by the newer and more potent P2Y_12_ inhibitors, prasugrel and ticagrelor. While they exhibit less variability, they are substantially more expensive and their increased potencies compound the bleeding risk already associated with DAPT. Hence, the contemporary dilemma is that of balancing efficacy, over-efficacy, and economic burden. Moreover, as aspirin represents one-half of DAPT, standardizing its therapeutic benefit between patients is a crucial objective that has yet to be achieved. Herein, we review the mechanisms of non-response to the constituent medication of DAPT and explore emerging approaches for addressing the aforementioned issues surrounding their clinical use.

## P2Y_12_ Inhibitors

### Overview of P2Y_12_ Inhibitors

**Fig. 1:**
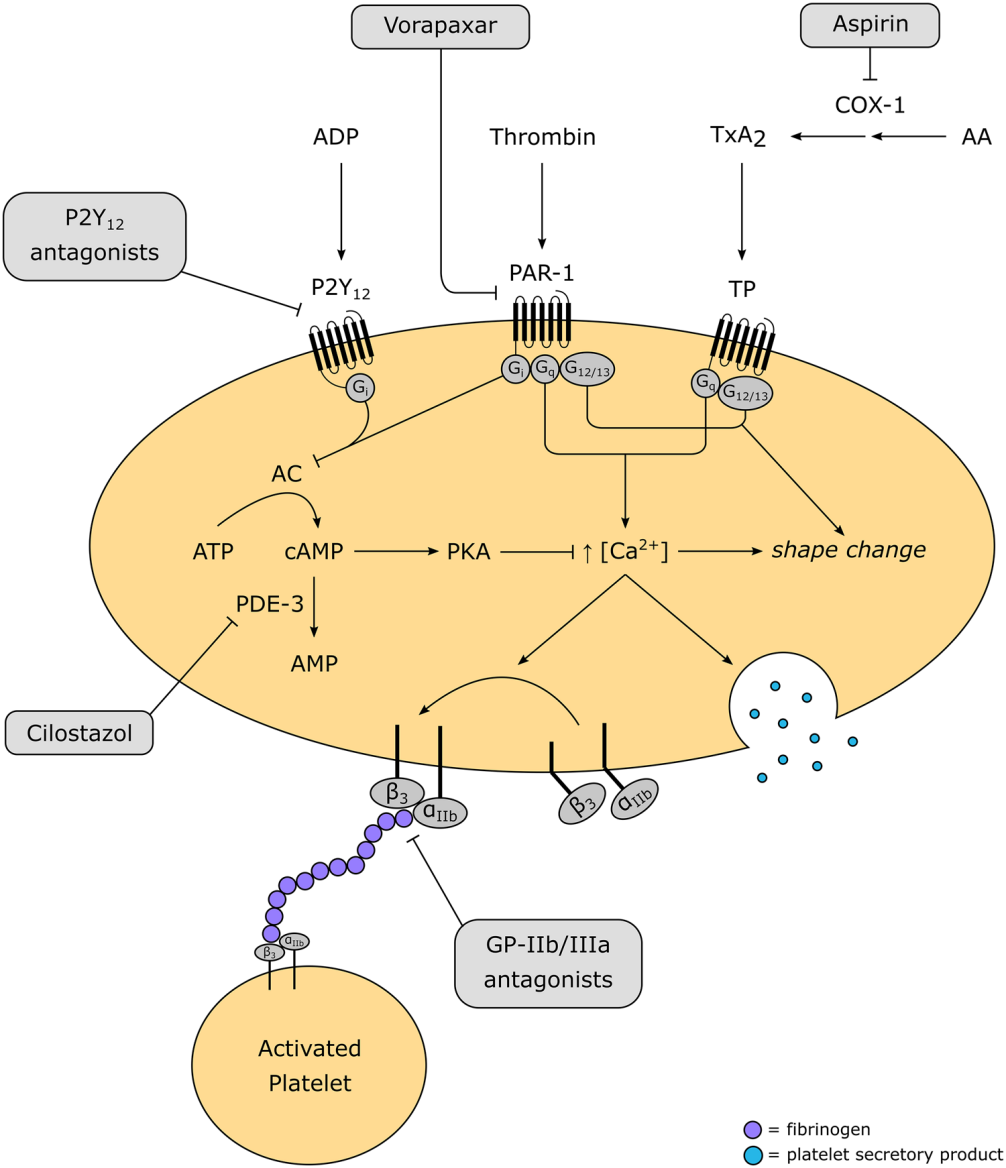
Overview of antiplatelet agent mechanisms. Platelets express a multitude of surface receptors that, when bound by their agonist, trigger intracellular signaling cascades leading to GPIIb/IIIa (fibrinogen receptor) conformational activation and platelet degranulation. Key drug targets include the G protein-coupled receptors P2Y_12_ (responds to ADP) and PAR-1 (responds to thrombin), the enzymes COX-1 and PDE-3, and the integrin GPIIb/IIIa. AA, arachidonic acid; AC, adenylate cyclase; ADP, adenosine diphosphate; AMP, adenosine monophosphate; ATP, adenosine triphosphate; cAMP, cyclic adenosine monophosphate; COX-1, cyclooxygenase-1; PAR-1, protease-activated receptor-1; PDE-3, phosphodiesterase-3; PKA, protein kinase A; TP, thromboxane receptor; TxA_2_, thromboxane A_2_

ADP is a soluble mediator of platelet aggregation that is released by damaged endothelial tissue and activated platelets. It acts in autocrine and paracrine fashions on the platelet membrane receptors, P2Y_1_ and P2Y_12_. Activation of the latter is integral for the amplification and maintenance of the local response, while the former has a more transient role in the initiation of ADP-dependent aggregation [6]. There are selective P2Y_1_ inhibitors in preclinical development [7, 8]; however, the only approved ADP-blocking antiplatelet drugs antagonize the P2Y_12_ receptor (Fig. 1). These drugs are typically combined with aspirin in DAPT for the management of ACS and secondary prevention of thromboembolic events (e.g., post-PCI). The superiority of DAPT over previous strategies, such as anticoagulation or aspirin alone, in preventing cardiovascular complications, has been demonstrated extensively [9, 10].

### Clopidogrel

**Fig. 2:**
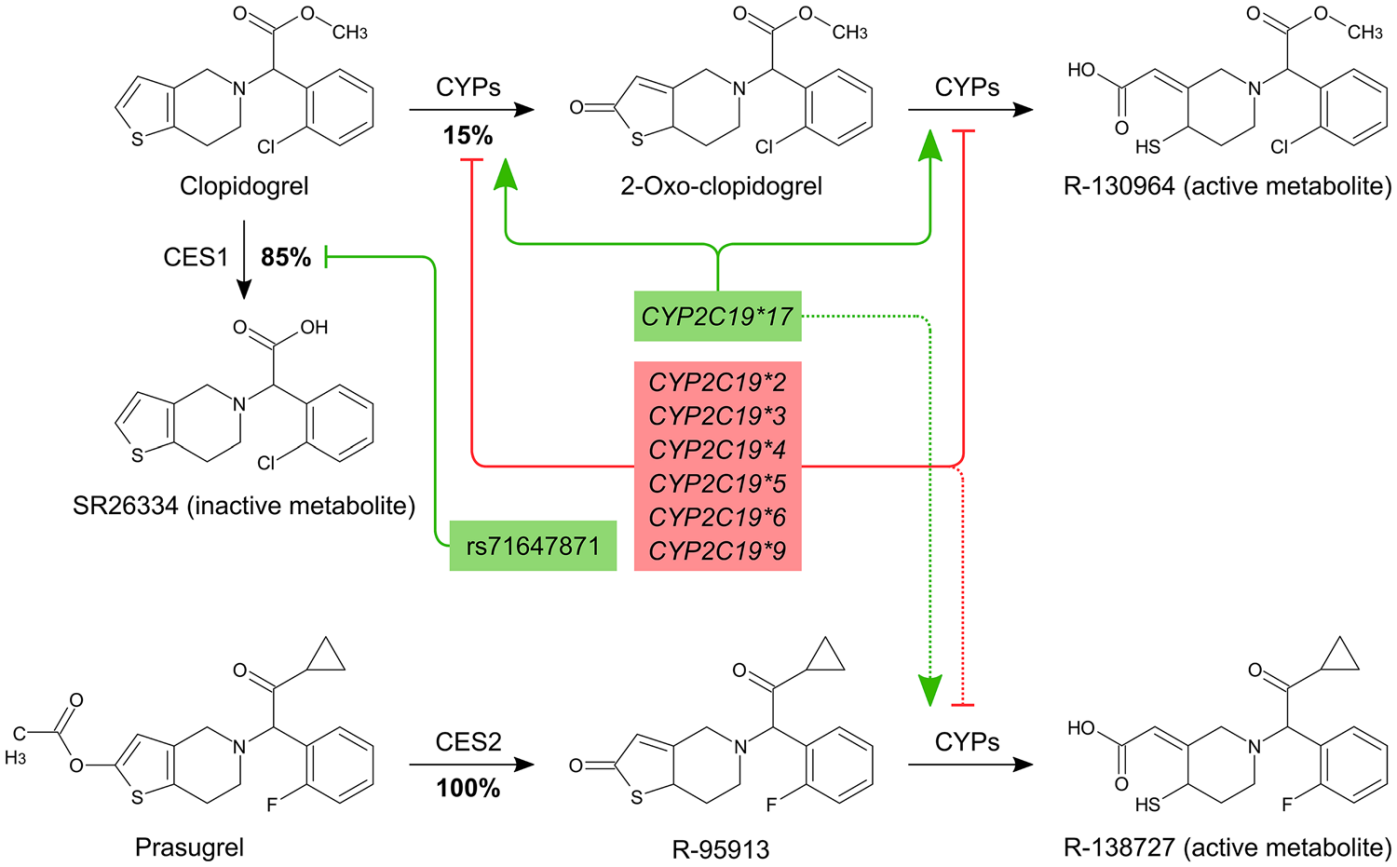
Metabolism of the thienopyridines. Clopidogrel is a prodrug, most of which (~ 85%) is inactivated by hepatic esterases (namely CES1), with the remainder undergoing oxidative bioactivation in two sequential steps catalyzed by multiple CYPs. Prasugrel is a prodrug that is activated in a two-step pathway catalyzed by intestinal esterases (namely CES2) and multiple CYPs respectively. Polymorphisms shown in red decrease drug exposure; those in green increase drug exposure. Despite CYP2C19 being involved in the bioactivation of both prodrugs, polymorphisms of the enzyme appear to have negligible effect on prasugrel’s efficacy (indicated by dotted lines). CES1, carboxylesterase-1; CES2, carboxylesterase-2; CYPs, cytochrome P450 enzymes

Despite clopidogrel’s blockbuster success, patient responsiveness is notoriously variable, with ~ 30% of patients exhibiting inadequate platelet inhibition [11]. Clopidogrel is a prodrug that is subject to transport by P-glycoprotein (P-gp). Most (~ 85%) of the administered dose is converted to an inactive carboxylic acid metabolite (SR26334) by hepatic esterases [12], with the remaining 15% undergoing two sequential oxidative steps to become activated (R-130964) (Fig. 2). Although the oxidative component of clopidogrel’s metabolism remains somewhat controversial [13], it is generally thought that CYP450 enzymes (CYPs) including CYP2C19, CYP3A4/3A5, CYP1A2, CYP2B6, and CYP2C9 are involved [14].

The PAPI study of 429 healthy Amish people originally estimated the heritability of clopidogrel’s inter-individual variability at 73% [15]. The authors reported CYP2C19*2, a loss-of-function (LoF) allele, to be strongly associated with ADP-induced platelet aggregation, exhibiting a dose–response relationship, and estimated that this polymorphism accounted for 12% of the total variation in clopidogrel response. Clinical factors, including age, BMI, and lipid levels, accounted for another ~ 10% of the observed variation; however, with ~ 78% of the variability remaining unexplained, a cascade of new *CYP2C19* SNPs was subsequently reported. Of these, the majority are LoF alleles (e.g., *3/*4/*5/*6/*9), although one gain-of-function (GoF) allele (*17) has also been identified. Owing to the multitude of alleles and their codominant expression, a patient’s clopidogrel sensitivity will lie along a spectrum according to their ability to metabolize the prodrug (Table 1); however, most other variants are quite rare and cannot adequately compensate for the unexplained variability (Table 2). Many other genes have been implicated, albeit less successfully compared to *CYP2C19*. Of note, *CES1*, which encodes hepatic carboxylesterase-1, carries out the major inactivating metabolic step to a carboxylic acid metabolite (Fig. 2). The rs71647871 LoF variant significantly increases drug exposure [16] and has been assigned a 2B level of evidence on PharmGKB; however, its rarity diminishes its overall impact. Polymorphisms of the P2Y_12_ receptor itself have also garnered attention; in fact, it has been argued that the CYP2C19 genotype is not influential and is simply a biomarker of P2Y_12_ functionality, through a yet undiscovered link [13]. While such a linkage is unlikely given that the genes are on separate chromosomes, an independent pharmacodynamic effect of P2Y_12_ polymorphisms is certainly plausible and would provide an appealing explanation for the remaining variability. Unfortunately, multiple early attempts failed to identify any meaningful associations [17–19] and despite some rekindled interest, results have remained unconvincing. Thus, a pharmacokinetic mode of variability mediated by CYP2C19 is still presumed to be the case. Several early meta-analyses confirmed the association between LoF alleles and major adverse cardiovascular events (MACE) [20, 21]. Subsequent RCTs would then investigate escalation strategies, whereby inadequate genotype-predicted or functional response would prompt higher dosages or alternative therapy.

**Table 1:** Predicted genotype–phenotype relationships for selected CYP2C19 variants

Phenotype	Rapid metabolizer	Normal metabolizer	Intermediate metabolizer	Poor metabolizer
Genotype	*17/*17 *1/*17	*1/*1 *2/*17 *3/*17 *4/*17 *6/*17	*1/*2 *1/*3 *1/*4 *1/*6	*2/*2 *2/*3 *2/*4 *2/*6 *3/*3 *3/*4 *3/*6 *4/*4 *4/*6 *6/*6

**Table 2:** Allelic frequencies for selected CYP2C19 variants

	European	East Asian	African American/Afro-Caribbean	Latino
*2	0.1469	0.2835	0.1815	0.1042
*3	0.0016	0.0725	0.0028	0.0008
*4	0.0024	0.0002	0	0.0005
*6	0.0003	0.0006	0	0
*17	0.2154	0.0205	0.2072	0.1666

Data sourced from PharmGKB (https://www.pharmgkb.org/page/cyp2c19RefMaterials)

Despite high on-treatment platelet reactivity (HTPR) being strongly associated with poor outcomes, RCTs examining the effects of PFT-guided escalation have produced mixed results. The GRAVITAS trial enrolled patients with HTPR post-PCI and randomized them to double-dose or standard-dose clopidogrel with no difference in the observed rate of MACE [22]. However, as HTPR spontaneously resolved in 38% of patients randomized to conventional therapy, probably due to transiently increased platelet reactivity following PCI, many patients may have been falsely classified as poor responders, thereby obfuscating the benefit of double-dose clopidogrel. Hence, the optimal timing and interpretation of PFTs post-PCI has been debated [23]. The authors also speculated that double-dose clopidogrel may have been insufficient in many cases, especially in CYP2C19*2 homozygotes. In the ARCTIC trial, patients were recruited prior to scheduled PCI and randomized to PFT-guided or conventional treatment and, again, no significant difference was observed [24]. However, the study received some criticism regarding the PFT used, the apparent low-risk population enrolled, the underpowered design and insufficient treatment escalation [25, 26].

Notable RCTs that instead used a genotype-guided approach include the IAC-PCI study [27] and the TAILOR-PCI study [28]. In contrast to those previously discussed, these trials adjusted therapy more aggressively in response to LoF genotypes. The former doubled the dose of clopidogrel for intermediate metabolizers and additionally added cilostazol for poor metabolizers, reporting a significantly lower rate of MACE in guided therapy (2.66% vs. 9.03%; *p* < 0.01). The latter study simply gave ticagrelor to patients with any LoF alleles and reported a nearly significant reduction in MACE (4.0% vs. 5.9%; HR, 0.66 [95% CI, 0.43–1.02]; *p* = 0.06), but the investigators noted that many subjects received newer-generation stents and had lower-than-anticipated event rates, which was not accounted for in their power calculation.

The recent GIANT study was a non-randomized, multicenter investigation of 1,445 patients with ST-elevation myocardial infarction (STEMI) who underwent successful PCI [29]. Maintenance therapy optimization (prasugrel or double-dose clopidogrel) in LoF carriers led to similar outcomes when compared to non-carriers, while non-optimized carriers had a ~ fivefold higher rate of complications (15.6% vs. 3.3%; *p* < 0.05). However, the non-optimized carriers represented a relatively small proportion of the total sample size and the decision to not modify treatment was at the discretion of the treating physicians, making the impact of unknown confounders an important consideration. Nevertheless, these results mirrored those in a prior study of a similar design [30] that reported a significantly increased risk of MACE in patients with LoF alleles without treatment adjustment (HR, 2.26 [95% CI, 1.18–4.32]; *p* = 0.013), supporting the argument for therapies guided by CYP2C19 status.

### Prasugrel

Prasugrel solved many of the issues encountered with clopidogrel by improving upon its pharmacokinetics. Firstly, unlike clopidogrel, there is no deactivating pathway for the parent drug; hence, ~ 100% is converted to active metabolite beginning with the formation of R-95913 by intestinal esterases (CES2) (Fig. 2) [31]. Consequently, prasugrel’s *apparent* potency is substantially higher than clopidogrel’s despite their active metabolites having comparable receptor affinities. Furthermore, with only 1 CYP-dependent step, prasugrel’s metabolic pathway is subject to less variability (Fig. 2) [31, 32]. Finally, prasugrel’s absorption is not hindered by the efflux transporter, P-gp [32]. Nevertheless, a small body of evidence of HTPR in the setting of prasugrel treatment would suggest that some individuals experience a subtherapeutic response [33], perhaps due to the highly polymorphic CYPs. However, despite prasugrel being activated by many of the same CYP isoforms as clopidogrel, a key meta-analysis found no significant associations between common polymorphisms and prasugrel response [34]. Indeed, in contrast to clopidogrel, patients with CYP2C19*2 LoF alleles respond strongly to prasugrel [35]. Aside from CYPs, various polymorphisms of the platelet endothelial aggregation receptor-1 (PEAR1), a transmembrane receptor that may also be involved in aspirin resistance, have been implicated in affecting prasugrel’s response, but the data are limited to PFTs and no clear clinical significance has been established [36, 37].

### Ticagrelor

Whereas clopidogrel and prasugrel are thienopyridines, ticagrelor is structurally distinct and does not require metabolic activation to exert its antithrombotic effects; however, its active and equally potent metabolite, AR-C124910XX, is formed by the action of CYP3A4/3A5 [38]. Intestinal uptake of ticagrelor might involve the organic anion transporter protein-1B1 (OATP1B1; encoded by *SLCO1B1*), while P-gp might be involved with its efflux, although little is currently known [39]. Ticagrelor and its metabolites may be hepatically eliminated via glucuronidation, mediated by multiple UDP-glucuronosyltransferase isoforms (encoded by *UGT*) [40]. A genome-wide association study (GWAS) identified three genes associated with the pharmacokinetics of ticagrelor: *SLCO1B1*, *UGT2B7*, and *CYP3A4* [41]; however, only modest effects were observed and no implications on safety or efficacy were noted. Yet, another study found no effect of *CYP3A4/3A5* or *SLCO1B1* genotype [42], although a subsequent study implicated a separate variant of *CYP3A4* (rs35599367) to markedly impair ticagrelor elimination [43]. More recently, Nie et al. analyzed associations between 35 variants (not including *CYP3A4* rs35599367) across 10 genes in a study of 68 healthy Chinese volunteers [44]. They found no association with *SLCO1B1*, *UGT2B7*, or *CYP3A4/3A5* genotype but reported homozygotes of the *CYP4F2* rs2074900 variant, commonly found in East Asian populations, to substantially increase peak drug concentration and total drug exposure. This is supported by the finding that the CYP4F2 genotype, albeit a separate variant (rs3093135), is associated with decreased on-treatment platelet reactivity and increased bleeding risk in patients on DAPT that includes ticagrelor [45, 46]; however, the mechanism of this interaction remains unclear.

### Current Status/Future Directions

**Fig. 3:**
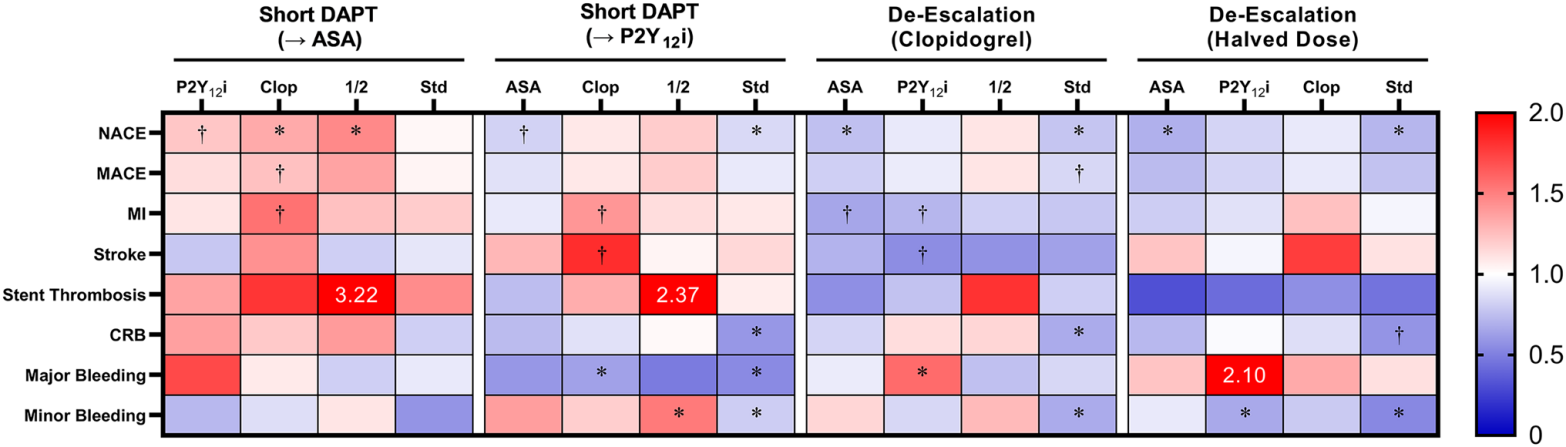
Summary comparisons of DAPT de-escalation strategies. The relative risk of selected outcomes (vertical axis) across four modified DAPT are shown via color mapping. Comparisons are made as top horizontal labels (large font) versus corresponding labels directly below (small font). Relative risk values outside the legend range are displayed directly in the cell. Data is derived from a network meta-analysis by Laudani et al. [56]. ASA, short DAPT (→ ASA); Clop, de-escalation (clopidogrel); CRB, clinically relevant bleeding; MACE, major adverse cardiac events; MI, myocardial infarction; NACE, net adverse cardiac events; P2Y_12_i, short DAPT (→ P2Y_12_i); Std, standard DAPT; ½, de-escalation (halved dose). *, statistically significant; †, borderline non-significant

Escalation strategies are becoming less relevant as current guidelines [47, 48] now recommend DAPT with routine use of potent P2Y_12_ inhibitors in the setting of ACS. The TRITON-TIMI 38 trial demonstrated the superiority of prasugrel to clopidogrel in PCI patients, especially with respect to periprocedural complications [49]; however, a significant increase in major bleeding events was noted, with some high-risk groups (e.g., advanced age, low bodyweight) failing to achieve a net clinical benefit. Similarly, the PLATO trial demonstrated increased bleeding risk with ticagrelor *vs*. clopidogrel [50]. Nevertheless, in an acute setting (i.e., ACS), using the less variable agents is prudent to maximize response likelihood. Furthermore, potent platelet inhibition is particularly important within the first few weeks of PCI, when stents have yet to fully endothelialize and the risk of thrombosis is greatest. However, while ischemic risk gradually declines, the bleeding risk remains relatively stable and eventually supersedes that of ischemia in the later phases of DAPT. Thus, de-escalation strategies that attenuate P2Y_12_ inhibition by an eventual switch to clopidogrel or dose reduction of either prasugrel or ticagrelor have been explored. Importantly, the former should be a guided process, whereby genotype pre-emptively predicts clopidogrel sensitivity or functional testing confirms an adequate response upon changing regimens. In the absence of a resistant phenotype, clopidogrel is apparently just as effective as its newer counterparts, which is suggested by the findings of Pereira et al. who concluded that the increased efficacy of prasugrel and ticagrelor is dependent on the carriage of CYP2C19 LoF alleles [51], and further supported by the results of the POPular Genetics trial that found genotype-guided de-escalation to clopidogrel to be noninferior to routine treatment with either prasugrel or ticagrelor [52]. A strategy of unguided de-escalation to clopidogrel in ACS patients has also been investigated [53–55], but this raises concern for those patients who are unresponsive to clopidogrel and should therefore be used cautiously, if at all. De-escalation can also be performed by reducing the duration of DAPT (short DAPT), which involves an early transition to monotherapy via termination of either aspirin (short DAPT → P2Y_12_i) or the P2Y_12_ inhibitor (short DAPT → ASA). All the aforementioned de-escalation approaches should, in theory, reduce the burden of bleeding; however, each has its drawbacks, and debate remains over which method is ideal. Recently, some thought-provoking evidence has emerged from a network meta-analysis of 29 studies (*n* = 50,602) by Laudani et al. that indirectly compared these strategies, primarily in ACS patients [56]. Key results are summarized in Fig. 3 and the studies [53–55, 57–82] that were included in their analysis are summarized in Supplemental Table 1. Compared to standard DAPT, all strategies besides short DAPT (→ ASA) significantly reduced minor bleeding and net adverse cardiovascular events (NACE – i.e., MACE + bleeding events). Both short DAPT (→ P2Y_12_i) and de-escalation to clopidogrel significantly reduced clinically relevant bleeding (minor + major bleeding), while de-escalation via halved dose exhibited a borderline non-significant reduction. Importantly, only short DAPT (→ P2Y_12_i) significantly reduced *major* bleeding (relative risk [RR], 0.54 [95% CI, 0.43–0.67]) and, when indirectly compared to the other strategies, demonstrated superiority over de-escalation to clopidogrel alone. Nevertheless, de-escalation to clopidogrel tended to outperform short DAPT strategies in ischemic outcomes, with nearly significant reductions in myocardial infarction (vs. short DAPT [→ ASA] and short DAPT [→ P2Y_12_i]) and stroke (vs. short DAPT [→ P2Y_12_i]). Overall, these data suggest that all the considered strategies, with the exception of short DAPT (→ ASA), produce the intended result of reduced bleeding events; however, only short DAPT (→ P2Y_12_i) achieved this for major bleeding specifically. Yet, this latter finding is balanced by an apparent inferiority versus de-escalation to clopidogrel with respect to ischemic outcomes. Indeed, with only a fraction of the full therapeutic duration, it stands to reason that short DAPT strategies will benefit from the greatest reductions in bleeding, at the cost of more ischemic events down the line. This reciprocal nature makes ascribing a clear winning strategy a difficult task, necessitating further studies with direct, head-to-head comparisons. Moreover, there are other pertinent considerations including strategy complexity and cost. De-escalation to clopidogrel, even if guided, is considerably cheaper than the other strategies; however, guided therapy is inherently more complex. Hence, the benefits of any DAPT strategy should be taken in the context of both economic burden and clinical feasibility.

**Table:** 

*Is There a Need for Precision Medicine?* ***P2Y***_***12***_*** Inhibitors***	
The newer, more potent P2Y_12_ inhibitors, prasugrel and ticagrelor, exhibit minimal inter-subject variability and achieve higher rates of therapeutic response in the general population; hence, there is a minimal clinical need to personalize their dosing regimens. Nevertheless, some research groups have identified certain alleles associated with an increased bleeding risk that should be investigated further. However, the most useful application of personalization appears to be that of DAPT de-escalation in ACS patients treated with PCI, the specific approach of which might be chosen according to clinical risk profile. Induction of DAPT with the newer agents is the most practical approach to preventing early post-procedural thrombosis, especially given that guided therapy cannot feasibly be implemented emergently, but long-term maintenance therapy with conventional DAPT is associated with excess bleeding. The two most promising strategies for combating this are de-escalation by the eventual downgrading of P2Y_12_ inhibition to clopidogrel and shortening the duration of DAPT via early discontinuation of aspirin. The latter appears to provide superior bleeding protection while the former appears to provide superior ischemic protection, findings which are unsurprising given their inherent differences. Nevertheless, both approaches reduce bleeding and their relative differences in reducing ischemic vs. hemorrhagic endpoints might be strategically utilized according to clinical context. For instance, in those with a high (e.g., previous ACS, bifurcation stent) or normal risk of ischemia, guided de-escalation to clopidogrel may be favorable, especially given its cost-effectiveness. Of course, this necessitates that the patient be sensitive to clopidogrel (i.e., devoid of major CYP2C19 LoF alleles); otherwise, the continuation of standard DAPT might be necessary. Alternatively, short DAPT with eventual transition to ticagrelor monotherapy may be favorable in those with high bleeding risk (e.g., prior bleeding, low bodyweight)	

## Aspirin

Aspirin is a widely used analgesic, antipyretic, and anti-inflammatory medication. Its more recent role in atherosclerotic cardiovascular disease (ASCVD) is attributed to its selective effects on anucleate cells (i.e., platelets) that are incapable of regenerating the COX-1 that aspirin irreversibly inhibits, thereby depleting them of the potent activator of aggregation, thromboxane A_2_ (TxA_2_) (Fig. 1) [83].

**Fig. 4:**
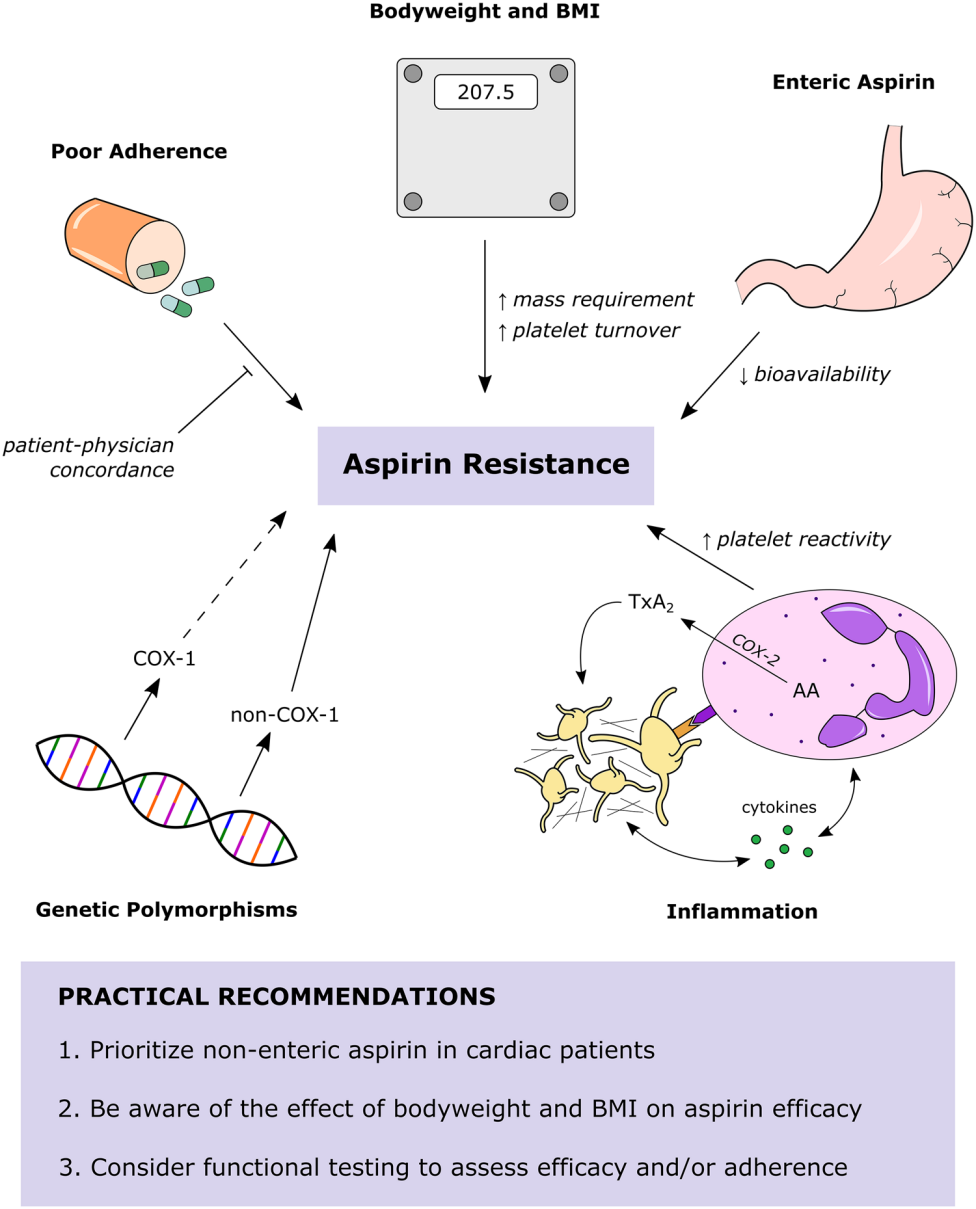
Likely modes of aspirin resistance. Poor response to aspirin may be caused by a multitude of factors, one or more of which may be relevant to any particular patient. Of these, the most important appear to be: (**a**) poor adherence, (**b**) insufficient dosing according to bodyweight and/or BMI, (**c**) use of enteric-coated aspirin with reduced bioavailability, (**d**) comorbid inflammatory states that alter platelet reactivity, and (**e**) polymorphisms of COX-1 that produce pharmacological resistance (relatively less likely) or other genes (non-COX-1) that affect baseline platelet reactivity (relatively more likely). AA, arachidonic acid; BMI, body mass index; COX, cyclooxygenase; TxA_2_, thromboxane A_2_

Considering its widespread use for a number of indications, including ACS, a concerning degree of aspirin non-response has been reported [84, 85], with some prevalence estimates reported at upwards of 50% [86]. However, available estimates are highly variable, owing in large part to discordance between attempts to define it. In a meta-analysis of 42 studies, Hovens et al. reported a weighted mean prevalence of laboratory-defined aspirin resistance of 24% (range = 0–57%) with an inter-study heterogeneity (*I*^*2*^) of 94% [85]. Most included studies used one of three PFTs to define aspirin non-response, the choice of which strongly influenced estimates, consistent with studies that have demonstrated poor agreement between PFTs [87, 88]. While this limits the practical applications of PFTs, they still provide reasonable evidence for aspirin resistance. The most specific test for the pharmacodynamic effect of aspirin is the measurement of serum thromboxane B_2_ (TxB_2_), a stable metabolite of TxA_2_ whose levels would be expected to be low in responders and high in non-responders. Interestingly, the prevalence of high TxB_2_ in aspirin-treated patients appears to be exceptionally low [89–91], suggesting that aspirin resistance, as measured by PFTs, is largely due to non-COX-1-mediated mechanisms (Fig. 4).

### Pseudo-resistance

Enteric-coated aspirin, used to mitigate gastrointestinal complications, has been frequently implicated in aspirin resistance [92, 93]. This “pseudo-resistance” has been substantiated by evidence for impaired bioavailability of enteric aspirin compared to its immediate-release counterpart [94–96], with individuals of higher bodyweight being particularly susceptible to treatment failure with the enteric-coated formulation [93]. Aspirin (acetylsalicylic acid [ASA]) is an acid and is therefore neutral at gastric pH, facilitating its absorption. In contrast, enteric aspirin is absorbed in the gut with near-neutral pH where aspirin exists in an ionized form with reduced absorption.

Poor adherence has also emerged as a potentially significant contributor to aspirin pseudo-resistance. In multiple case series, the majority of aspirin non-responders were found to be non-adherent and were confirmed to be aspirin-sensitive after controlled administration [97, 98]. In fact, many studies have shown non-adherence to be one of the most important factors in the apparently suboptimal response to aspirin [99, 100]. Clearly, strategies to improve adherence are warranted and represent a simple step to mitigate population-wide treatment failure.

### Bodyweight and BMI

A 2018 meta-analysis of 10 trials (*n* = 117,279) on aspirin in primary prevention found that low-dose aspirin (75–100 mg) reduced the risk of MACE in those of lower weight (50–69 kg) only and not in those weighing 70 kg or more (HR, 0.75 [95% CI, 0.65–0.85] vs. HR, 0.95 [95% CI, 0.86–1.04]), with any benefit in the higher bodyweight cohort observed only at higher doses (≥ 325 mg) [101]. Interestingly, the authors noted diminished protective effects of aspirin against colorectal cancer (CRC) in those of higher bodyweight treated with low-dose aspirin, which was again rescued by higher doses. It is thought that increased BMI attenuates COX-1 inhibition via increased platelet activity and turnover [101]; however, the loss of protection against CRC, a COX-2-dependent effect [102], implies the resistance conferred by higher bodyweight is not exclusive to platelets. Although platelet turnover probably contributes, especially in patients with elevated BMI, it is also likely that higher bodyweight requires adjustment in line with simple variations in blood volume, even in the absence of an elevated BMI. Given that guidelines do not recommend weight-adjusted dosing, it is likely that insufficient dosing is a substantial contributor to treatment failure, especially considering the average American male adult weighs 90 kg. An early meta-analysis showed that 75 mg aspirin was the lowest protective dose in ASCVD [103]; however, 75 mg enteric aspirin has similar bioavailability to 50 mg non-enteric aspirin [96]. Notably, the original aspirin studies were performed a few decades ago using non-enteric aspirin in a population that had a lower average bodyweight. Today, with the widespread use of enteric aspirin, it is unsurprising that patients with higher bodyweights will receive inadequate exposure to the drug.

### Inflammation

Inflammation is a cardinal feature of many disease processes, including non-obvious examples like obesity and ASCVD [104]. Moreover, inflammation and thrombosis are intimately linked through a variety of mechanisms collectively referred to as thromboinflammation [105]. At the simplest level, TxA_2_ may be alternatively synthesized via COX-2, whose expression is predominantly induced under inflammatory conditions. Accordingly, COX-2 is conventionally thought to be expressed by immune cells, but it may also be expressed in platelets, and both of these additional sources of TxA_2_ represent an increased aspirin requirement for adequate suppression of thrombotic activity [106]. Moreover, beyond simply releasing a variety of pro-inflammatory mediators, platelets also respond to them, enhancing their activation [107]. Platelets are also capable of directly interacting with immune cells, often mediated by activated integrin complexes under inflammatory conditions. One such complex, CD11b/CD18, can interact with the GPIbα component of the von Willebrand factor (vWF) receptor [108] to not only promote leukocyte effector functions [108] but also contribute to platelet outside-in-signaling, leading to GPIIb/IIIa (fibrinogen receptor) activation and degranulation of secretory products, including numerous cytokines from α-granules [105]. Conversely, inflammation may also impart anti-thrombotic effects, one important example being C-reactive protein (CRP), which Brennan et al. demonstrated to be capable of antagonizing GPIIb/IIIa [109]. Moreover, even low-grade inflammatory states such as obesity have been strongly associated with an elevated CRP [110]. Thromboinflammation comprises a complex set of processes, with both pro- and anti-thrombotic components; if the net effect leans in favor of the former, this could represent an important mode of resistance in patients with comorbid inflammatory states, including the many patients who take the medication for secondary prevention.

### Pharmacogenomics

Polymorphisms relevant to aspirin can principally be categorized as those that affect COX-1 directly, and those with non-COX-1-dependent effects that nonetheless influence platelet activity. While many have been investigated, insufficient replicability and small study size have been prominent issues, and the meta-analyses of the representative studies remain unconvincing. In 2008, Goodman et al. conducted a systematic review of 31 studies and analyzed the pooled data of five commonly implicated genes in aspirin resistance: *ITGB3*, *ITGA2*, *PTGS1*, *P2RY12*, and *P2RY1* [111].

*ITGB3* is the gene encoding the β_3_ subunit of GPIIb/IIIa and a total of 10 studies (*n* = 968) were included for analysis of the Pl^A1/A2^ genotype. A moderate-to-high inter-study variability was reported (*I*^*2*^ = 63.1%) and a significant association with resistance was found only in a sub-analysis of healthy individuals (odds ratio [OR], 2.36 [95% CI, 1.24–4.49]; *p* < 0.01). The authors speculated that diseased individuals were more likely to be taking medications that could obscure a relationship in the data by influencing platelet reactivity. Importantly, the authors also emphasized the influence of PFT used to define aspirin resistance on the results.

*ITGA2*, *PTGS1*, *P2RY12*, and *P2RY1* are the genes encoding the GPIa component of the GPIa/IIa collagen receptor, COX-1, and the platelet ADP receptors P2Y_12_ and P2Y_1_, respectively. No associations were observed with any of the variants analyzed; however, it should be noted that considerably fewer studies were available to analyze these gene effects.

These results suggest that the heritable aspects of aspirin resistance may be more complex than singularly considered SNPs. For instance, while the meta-analysis by Goodman et al. found no significant association with any solitary SNP of the COX-1 gene (*PTGS1*), Maree et al. identified an aspirin-resistant COX-1 *haplotype* [112]. Another complicating factor in studying aspirin resistance is its frequent co-administration with other antiplatelet medications, making it difficult to determine if a phenotype is attributed to effects on aspirin, the other antiplatelet, or both. In a sub-study of the OPUS-TIMI 16 trial, Maree et al. identified a bleeding phenotype associated with a variant of the gene coding for GPIIb/IIIa (*GNB3*) and attributed it to modulatory effects on the GPIIb/IIIa inhibitor used in the trial, orbofiban [113]; however, as all of the patients included were also on aspirin, whose TxA_2_-dependent pathway also interacts with GPIIb/IIIa, one has to conclude that its efficacy may also have been altered.

Recently, other gene candidates have garnered attention. For instance, a common variant of the serotonin transporter (*5-HTT*), one of whose role is to take up serotonin into platelets for storage in dense granules, appears to attenuate the response to aspirin by increasing basal platelet activity [114]. Another notable candidate is *PEAR1*, which encodes a platelet transmembrane protein that is thought to stabilize thrombi in response to platelet-to-platelet contact [115]. A variety of SNPs have been described, with some having been associated with platelet reactivity [116–118] and/or cardiovascular events [118]. As previously mentioned, insufficient inhibition of COX-1 by aspirin appears to be extremely rare (as suggested by serum TxB_2_). Thus, auxiliary pathways, such as those involved with *5-HTT* and *PEAR1*, may contribute to resistance in some patients by affecting baseline platelet function; however, studies remain limited, and more work is required to elucidate their roles in thrombosis and patient outcomes.

### A Note on Aspirin in Primary Prevention

Current guidelines do not support the use of aspirin in primary prevention as a prophylactic benefit has not been demonstrated to outweigh the associated risk of major bleeding in patients presumed to be at high-risk of ASCVD. In a recent article, Cofer et al. lay out a rational argument for a new approach to aspirin in primary prevention [119]. They point to three key RCTs (ASCEND [120], ARRIVE [121], and ASPREE [122]) whose results paved the way for the current guidance to avoid aspirin in primary prevention. These trials enrolled patients with cardiovascular risk factors (e.g., diabetes, advanced age); however, event rates were low and inconsistent with populations that would truly be considered high-risk. Furthermore, the authors contrast aspirin with how anti-hypertensive and statin therapies are used for primary prevention of ASCVD—the latter two may be prescribed on the basis of a high cardiovascular risk profile in combination with suggestive biomarker findings (elevated blood pressure and low-density lipoprotein cholesterol [LDL-C], respectively), while studies on aspirin in primary prevention have only considered risk factors. Thus, the “missing link” for the successful use of aspirin in primary prevention may be the lack of biomarker use (i.e., PFTs) to more precisely determine eligibility for primary prophylactic aspirin. Another important point made by the authors is that both the benefit (antithrombosis) and risk (bleeding) associated with aspirin are functions of platelet activity, making PFTs a logical strategy to identify patients who are more likely to achieve a net clinical benefit. Of course, as previously discussed, there are many different PFTs that are poorly standardized and yield incongruent results. Cofer et al. similarly recognized this fact but argue for the use of light transmission aggregometry (LTA), widely considered to be the gold standard PFT, for assessing baseline platelet activity in patients considered to be at high-risk of ASCVD. As a rationale, they explain that when LTA is used to assess baseline platelet activity in a population, a bimodal distribution is produced that can reasonably distinguish those with low platelet reactivity from those with platelet hyperactivity, a result that is reproducible across multiple agonists and over time.

**Table:** 

*Is There a Need for Precision Medicine?* ***Aspirin***	
The pharmacogenomics of aspirin non-response are poorly elucidated and appear to be of relatively minimal concern compared to other, non-genetic factors. Enteric aspirin has reduced bioavailability and does not have sufficient evidence to support its use in ASCVD; thus, it should be avoided in the cardiac patient, although guidelines do not always explicitly make this point [123]. With respect to personalization, the most useful application appears to be that of dosing according to bodyweight and/or BMI. The common low-dose aspirin strategy is aimed at achieving the minimum protective dose such that the risk of bleeding is lessened. However, this leaves minimal “buffer,” putting many non-average patients at risk of failing to reach the threshold concentration required to achieve benefit, which can be due to any number of factors, including body size and composition [124]. Moreover, the studies from which this approach is derived are decades old and took place when the prevalence of obesity was much lower. Recent evidence suggests that only patients of lower bodyweight benefit from the cardioprotective effects of low-dose aspirin. In contrast to patients on warfarin who receive functional testing to verify the adequate response, aspirin response is not routinely tested for and is simply presumed to be conferring a benefit. Considering the pervasiveness of ASCVD and the widespread use of aspirin in its treatment, this represents a considerable issue that could potentially be remedied through standardized dosing strategies. Furthermore, non-adherence appears to be especially relevant to aspirin, which may be a consequence of the patient belief that aspirin is less important than other medications, given its routine household use for minor indications and the ability to acquire it without a prescription. Whatever the reason, non-adherence is likely a sizeable contributor to treatment failure, necessitating appropriate intervention. Inflammation is another important consideration; common afflictions, including obesity and ASCVD, constitute chronic inflammatory states and have all been associated with increased platelet reactivity [125, 126]. Thus, many patients, including those receiving aspirin for secondary prevention, may be predisposed to HTPR, and yet continue to be prescribed the same low-dose regimens as those without preexisting disease. All these factors can impact the holistic efficacy of DAPT and should not be overlooked. However, with evidence that short DAPT followed by P2Y_12_ inhibitor monotherapy is as effective as conventional DAPT but with less bleeding, there may be a move away from using aspirin post-PCI [127] Finally, the avoidance of aspirin in the primary prevention of ASCVD is likely a missed opportunity for clinicians. RCTs of aspirin in primary prevention have demonstrated an excess risk of bleeding, negating any potential benefit; however, inappropriate patient selection in these studies might underpin the disappointing results. Increased personalization with PFTs, such as LTA, might be better able to identify high-risk patients who are more likely to achieve a net clinical benefit from primary prophylactic aspirin	

## Conclusions

Antiplatelet agents are critical in the management of thrombotic disease, but their highly variable nature makes selecting the appropriate drug and dosing regimen a challenge. This is especially relevant in ACS patients treated with PCI who typically require 1 year of DAPT to prevent the feared complication of stent thrombosis. As bleeding and thrombosis exist on a continuum, DAPT inherently increases the risk of hemorrhage and, thus, partakes in a delicate balancing act that should ideally be tailored to patient-specific requirements. Two key methods employed by precision medicine are genetic and functional testing.

Functional testing would appear to be ideal as it gives a direct measure of the effectiveness of a specific drug regimen; however, there are multiple modalities and they have poor agreement. Furthermore, as many antiplatelet agents are metabolized by CYP enzymes, dietary factors and other drugs can impact their metabolism, creating temporal variability. Hence, a functional test can only indicate the effectiveness of a drug regimen at a particular point in time. The PCI procedure itself can transiently activate platelets such that a PFT performed soon thereafter would overestimate a patient’s thrombotic risk, making the timing and interpretation of such tests a challenge. Nevertheless, they can clearly prove patient adherence and, as non-adherence is a major contributor to clinical non-response, they can prompt intervention to address it. Aside from assessing treatment efficacy, functional testing may also be useful in risk-stratification, one prudent application being the quantification of baseline platelet reactivity to identify patients likely to benefit from primary prophylactic aspirin.

Genetic screening can also be used to guide therapy. While a number of polymorphisms have been associated with response to antiplatelet agents, this is complex as multiple polymorphisms are often implicated, each with a minor contribution. Moreover, in cases where a single pharmacogene appears to be dominant, there may be any number of polymorphisms with varying degrees of effect on therapeutic response; thus, predicting response according to a specific genotype is not trivial. This is effectively illustrated by clopidogrel and the numerous *CYP2C19* LoF alleles that have been identified.

In contrast to guided therapy, some companies have discovered newer agents with improved potency and much less variability, yet they are not without their limitations. They are significantly more expensive, which is particularly challenging as they are often used for a number of years, creating a significant economic burden. Furthermore, owing to their increased potencies, they are associated with greater rates of bleeding, which is the current dilemma with contemporary DAPT strategies that involve prasugrel or ticagrelor in lieu of clopidogrel. Whereas stent thrombosis was once of major concern, new-generation drug-eluting stents (DES) have substantially reduced its incidence, decreasing the benefit of intense antiplatelet regimens and highlighting their tendency to precipitate bleeding. As a potential solution, guided selection of P2Y_12_ inhibitors has been shown to be as effective as routine use of the more potent agents, but with a significantly lower bleeding risk [128]. Earlier investigations focused on escalation strategies, whereby potent P2Y_12_ inhibitors were reserved for clopidogrel non-responders. However, as the early post-procedure period after PCI with DES implantation is associated with the greatest risk of thrombosis, especially when neointimal coverage has yet to be achieved, potent platelet inhibition is preferred, whereas the later timepoints are amenable to guided de-escalation to clopidogrel as evidenced by attenuated bleeding risk without a trade-off in ischemic endpoints [129]. However, a non-guided approach of shortening the duration of DAPT has also been shown to reduce bleeding [56].

**Fig. 5:**
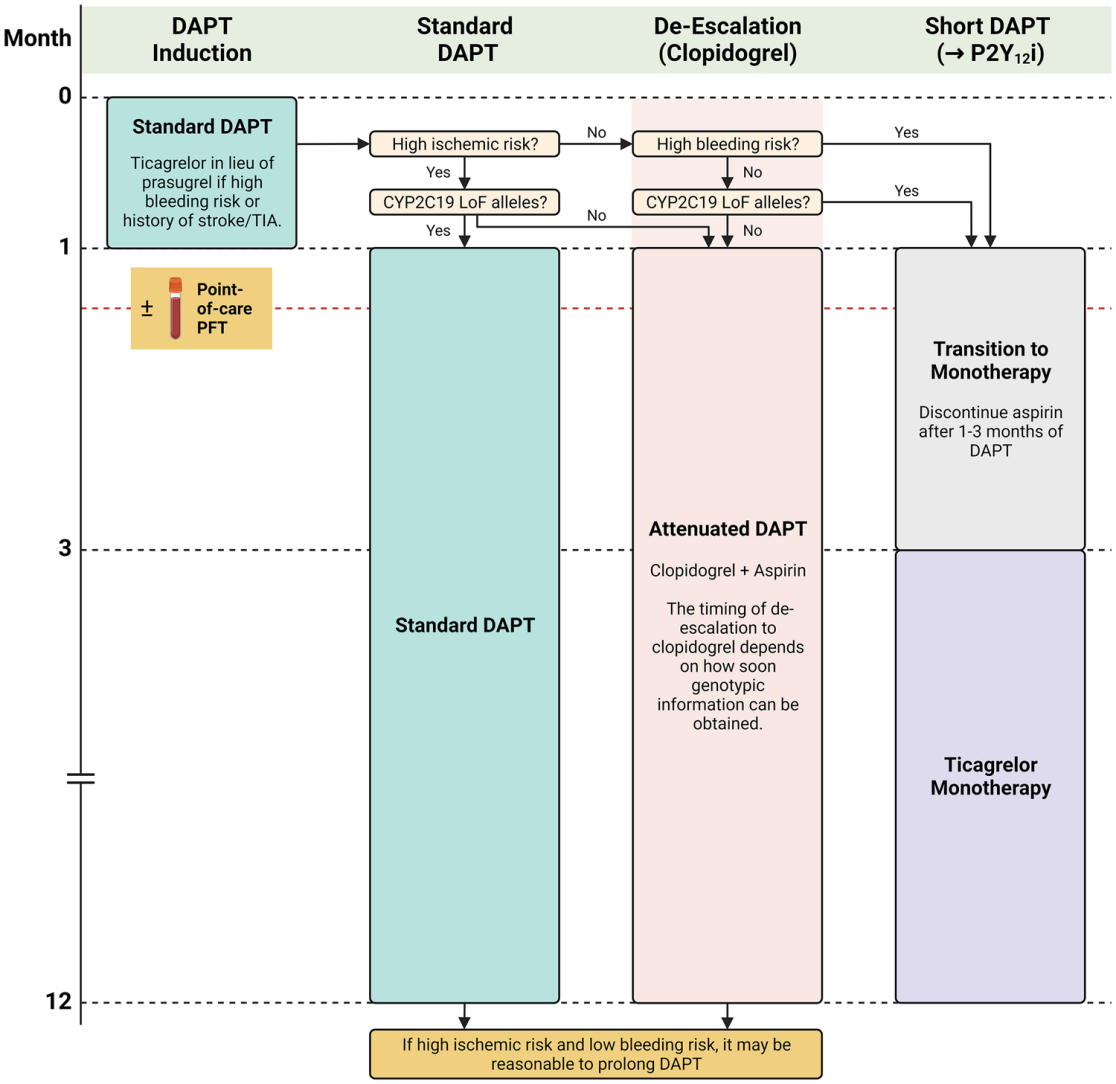
Practical DAPT de-escalation algorithm in patients with ACS treated by PCI. Patients presenting with ACS require periprocedural and maintenance DAPT. Initial treatment should include a high potency P2Y_12_ inhibitor (ticagrelor if high bleeding risk). In patients with high ischemic risk, de-escalation to clopidogrel can be considered if devoid of CYP2C19 LoF alleles; otherwise, standard DAPT should be maintained. In either case, prolongation of DAPT beyond 12 months can be considered on a case-by-case basis. In patients without high ischemic risk, high bleeding risk, nor LoF alleles, de-escalation to clopidogrel should be considered; if either of the latter two are present, short DAPT with transition to ticagrelor monotherapy after 1 to 3-months should be considered. In any approach, and at the discretion of the treating physician, periodic platelet function testing can be considered to confirm efficacy and/or adherence. ACS, acute coronary syndrome; DAPT, dual antiplatelet therapy; LoF, loss-of-function; P2Y_12_i, P2Y_12_ inhibitor; PFT, platelet function test; TIA, transient ischemic attack. Created with BioRender.com

In the search for the optimal DAPT strategy, a number of candidate approaches have been investigated. The two most promising, de-escalation to clopidogrel and short DAPT with eventual aspirin discontinuation, have been shown to mitigate excess bleeding without compromising anti-thrombotic efficacy; however, while their comparisons are limited to indirect evidence [56], the latter appears to provide superior bleeding protection while the former appears to provide superior ischemic protection. Furthermore, given that DAPT with clopidogrel is just as effective as DAPT with the newer P2Y_12_ inhibitors when patients are devoid of major CYP2C19 LoF alleles, it should be prioritized when possible to reduce economic burden. When taking these considerations together, a practical DAPT algorithm for ACS patients treated with PCI can be constructed (Fig. 5). Since genotypic information cannot be obtained emergently, patients presenting with ACS should be initiated on standard DAPT with prasugrel or ticagrelor to ensure therapeutic efficacy. Ticagrelor should be used in lieu of prasugrel in patients with higher bleeding risk (e.g., age ≥ 75 years, weight < 60 kg) or a history of stroke/transient ischemic attack [47]. Those with higher ischemic risk (e.g., prior ACS, bifurcation stents) and no major CYP2C19 LoF alleles could be de-escalated to attenuated DAPT with clopidogrel; if LoF alleles are present, standard DAPT should be continued. In either case, the decision to prolong DAPT beyond 12 months should be based on clinical bleeding risk and should be made on a case-by-case basis. In those without high ischemic risk, high bleeding risk, nor major LoF alleles, attenuated DAPT should be prioritized; if either of the latter two is present, short DAPT with the transition to ticagrelor monotherapy after 1 to 3 months should be considered. In any of these approaches, and at the discretion of the treating physician, periodic functional testing could be considered to confirm efficacy and/or adherence.

Finally, while much attention has been given to P2Y_12_ inhibition and the genetic influences that alter its efficacy in DAPT, non-genetic factors should not be overlooked. For instance, aspirin non-response can probably be largely explained by the triad of non-adherence, use of enteric formulations, and inappropriate dosing according to bodyweight and BMI. Given that aspirin represents half of DAPT, these issues should be addressed equally to ensure standardized DAPT efficacy.

## Supplementary Information

Below is the link to the electronic supplementary material.

Supplementary file1 (DOCX 24 KB)
