# Supplementary material for: Personalized Dual Antiplatelet Therapy in Acute Coronary Syndromes: Striking a Balance Between Bleeding and Thrombosis
Source: Curr Cardiol Rep. 2023 Jun 1;25(7):693–710. doi: 10.1007/s11886-023-01892-9 (PMC10307718; doi:10.1007/s11886-023-01892-9)
Supplement: Supplementary file 1 — Supplementary file1 (DOCX 24 KB) [file 11886_2023_1892_MOESM1_ESM.docx]

## **Supplementary Table 1. RCTs Investigating DAPT De-Escalation in ACS Patients Treated with PCI**

**Study Number of Patients Experimental Strategy Timing of De- Follow-Up**

**Randomized Escalation Period**

| ANTARCTIC (2016) | 877 | De-escalation to clopidogrel; PFT-guided | 1 month | 12 months |
| --- | --- | --- | --- | --- |
| TOPIC (2017) | 646 | De-escalation to clopidogrel; unguided | 1 month | 12 months |
| TROPICAL-ACS (2017) | 2,610 | De-escalation to clopidogrel; PFT-guided | 1 week | 12 months |
| Li et al. (2018) | 653 | De-escalation to clopidogrel; unguided | Variable | 12 months |
| POPular Genetics (2019) | 2,488 | De-escalation to clopidogrel; genotype-guided | <48 hours | 12 months |
| TALOS-AMI (2021) | 2,697 | De-escalation to clopidogrel; unguided | 1 month | 12 months |
| BLESS (2016) | 193 | De-escalation to half dose prasugrel; unguided | 1 month | 12 months |
| HOST-REDUCE-  POLYTECH-ACS (2020) | 2,338 | De-escalation to half dose prasugrel; unguided | 1 month | 12 months |
| HOPE-TAILOR (2021) | 120 | De-escalation to half dose prasugrel or ticagrelor; unguided | 1 month | 9 months |
| A-MATCH (2021) | 255 | De-escalation to half dose prasugrel; PFT-guided or unguided | Before discharge | 12 months |
| RESET (2012); ACS subgroup | 601 | Short DAPT to aspirin monotherapy | 3 months | 12 months |
| EXCELLENT (2012); ACS subgroup | 744 | Short DAPT to aspirin monotherapy | 6 months | 12 months |
| OPTIMIZE (2013); ACS subgroup | 996 | Short DAPT to aspirin monotherapy | 3 months | 12 months |
| ISAR-SAFE (2016); ACS subgroup | 1,601 | Short DAPT to aspirin monotherapy | 6 months | 15 months |
| I-LOVE-IT 2 (2016); ACS subgroup | 1,496 | Short DAPT to aspirin monotherapy | 6 months | 18 months |
| IVUS-XPL (2016); ACS subgroup | 686 | Short DAPT to aspirin monotherapy | 6 months | 12 months |
| ITALIC (2017); ACS subgroup | 806 | Short DAPT to aspirin monotherapy | 6 months | 24 months |
| NIPPON (2017); ACS subgroup | 1,075 | Short DAPT to aspirin monotherapy | 6 months | 18 months |
| DAPT-STEMI (2018) | 870 | Short DAPT to aspirin monotherapy | 6 months | 12 months |
| SMART-DATE (2018) | 2,712 | Short DAPT to aspirin monotherapy | 6 months | 18 months |
| REDUCE (2019) | 1,496 | Short DAPT to aspirin monotherapy | 3 months | 24 months |
| IDEAL-LM (2019); ACS subgroup | 305 | Short DAPT to aspirin monotherapy | 4 months | 24 months |
| ONE-MONTH DAPT  (2021); ACS subgroup | 1,192 | Short DAPT to aspirin monotherapy | 1 month | 12 months |
| MASTER DAPT (2021); ACS subgroup | 2,211 | Short DAPT to aspirin monotherapy or P2Y_12_i monotherapy | 1 month | 12 months |
| SMART-CHOICE  (2019); ACS subgroup | 1,741 | Short DAPT to clopidogrel monotherapy | 3 months | 12 months |
| STOPDAPT-2 ACS  (2022) | 4,136 | Short DAPT to clopidogrel monotherapy | 1 month | 12 months |
| GLOBAL LEADERS  (2019); ACS subgroup | 7,487 | Short DAPT to ticagrelor monotherapy | 1 month | 24 months |
| TWILIGHT (2020); ACS subgroup | 4,614 | Short DAPT to ticagrelor monotherapy | 3 months | 15 months |
| TICO (2020) | 3,056 | Short DAPT to ticagrelor monotherapy | 3 months | 12 months |
